# Molten‐Salt‐Mediated Chemical Looping Oxidative Dehydrogenation of Ethane with *In‐Situ* Carbon Capture and Utilization

**DOI:** 10.1002/cssc.202401473

**Published:** 2024-11-15

**Authors:** Kyle Vogt‐Lowell, Dennis Chacko, Kunran Yang, Jace Carsten, Junchen Liu, Matthew Housley, Fanxing Li

**Affiliations:** ^1^ Department of Chemical and Biomolecular Engineering North Carolina State University 911 Partners Way Raleigh, North Carolina 27695–7905 USA; ^2^ School of Engineering Newcastle University Merz Court Newcastle upon Tyne NE1 7RU United Kingdom

**Keywords:** CO_2_capture and utilization, Chemical looping, Molten salts, Ethane oxidative dehydrogenation

## Abstract

The molten‐salt‐mediated oxidative dehydrogenation (MM‐ODH) of ethane (C_2_H_6_) via a chemical looping scheme represents an effective carbon capture and utilization (CCU) method for the valorization of ethane‐rich shale gas and concurrent mitigation of carbon dioxide (CO_2_) emissions. Here, stepwise experimentation with Li_2_CO_3_‐Na_2_CO_3_‐K_2_CO_3_ (LNK) ternary salts (i) assessed how each component of the LNK mixture impacted ethane MM‐ODH performance and (ii) explored physicochemical and thermodynamic mechanisms behind melt‐induced changes to ethylene (C_2_H_4_) and carbon monoxide (CO) yields. Of fifteen screened LNK compositions, nine exhibited ethylene yields greater than 50 % at 800 °C while maintaining C_2_H_4_ selectivities of 85 % or higher. LNK salts rich in Li_2_CO_3_ content yielded more ethylene and CO on average than their counterparts, and net CO_2_ capture per cycle reached a maximum of ~75 %. Extended MM‐ODH cycling also demonstrated long‐term stability of a high‐performing LNK medium. Density functional theory (DFT) calculations and *ab initio* molecular dynamics (AIMD) simulations suggested that the molten salt does not directly activate C_2_H_6_. Meanwhile, an empirical model informed by experimental data and reaction thermodynamics adequately predicted overall MM‐ODH performance from LNK composition and provided insights into the system′s primary drivers.

## Graphical abstract

A sustainable alternative to steam cracking, the molten‐salt‐mediated oxidative dehydrogenation (MM‐ODH) of ethane (C_2_H_6_) in Li_2_CO_3_‐Na_2_CO_3_‐K_2_CO_3_ (LNK) couples thermal cracking with melt‐mediated CO_2_ capture to realize commercially competitive C_2_H_4_ and CO yields. LNK composition strongly influences MM‐ODH process outcomes. While Li_2_CO_3_‐rich media demonstrate the highest Y_C2H4_ and net CO_2_ capture capacities, increasing Na_2_CO_3_/K_2_CO_3_ content maximizes Y_CO_.

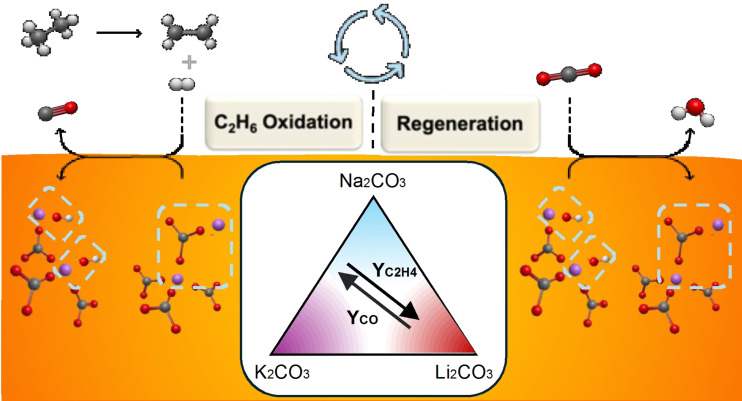

## Introduction

A worsening climate crisis coupled with a carbon‐centric global economy has led scientists to seek climate adaptation and mitigation strategies that marry urgency with socioeconomic practicality. Undoubtedly, to achieve net zero emissions by 2050 and avoid irreversible environmental damage, atmospheric CO_2_ concentrations must drop at unprecedented rates. With much time needed to sensibly phase out fossil fuels and little time left to substantially reduce emissions, carbon capture, utilization, and sequestration (CCUS) technologies have important roles to play.

One such role is the decarbonization of industries with stubborn emissions. Here, CCUS can provide relatively rapid pathways to low‐carbon‐intensity processes while minimizing disruptions to presently vital industries. While the sequestration of captured carbon into geological formations may offer thousand‐year CO_2_ retention times, current practical, regulatory, and financial uncertainties limit the approach′s near‐term practicality. Carbon capture and utilization (CCU) applications, however, convert captured CO_2_ into value‐added products,^1–9^ thereby offering both economic and environmental incentives to stakeholders. In low‐margin sectors with high‐emission processes, ensuring this alignment is paramount for realizing commercial‐scale emissions mitigation.

The steam cracking of ethane is a quintessential example of a capital‐intensive process within the petrochemical industry that stands to benefit significantly from CCU. The standard for ethylene production, steam cracking converts ethane to ethylene and hydrogen (H_2_) in fired tubular reactors that operate between 750 °C and 875 °C to overcome high reaction endothermicity (ΔH=144.53 kJ/mol at 827 °C).^10^ Short residence times (0.1–0.5 s) and rapid product quenching allow modern cracker furnaces to attain ethylene yields of 52–55 %.^10,11^ Despite its high energy demand (16 MJ/kg C_2_H_4_^11^), equilibrium limitations,^10–13^ and proclivity for reactor coking, steam cracking produces ethylene relatively cheaply, with an abundant supply of domestic shale gas reducing costs even further. However, ethylene produced in this way has a carbon intensity of ~1.5 metric tons CO_2_‐eq/metric ton C_2_H_4_ due to the process’ high operating temperature, complex downstream cryogenic separation, and associated shale gas flaring.^11,14^ The size of this footprint is second only to ammonia for large‐volume chemicals, so much effort has been devoted to exploring sustainable alternatives to this well‐optimized but environmentally unfriendly approach.

From a thermodynamic and sustainability standpoint, the oxidative dehydrogenation (ODH) of ethane represents a more favorable chemical pathway than thermal cracking. In this process, ethane and oxygen (O_2_) are co‐fed to a single reactor and react to form ethylene and steam. Water formation in place of hydrogen alleviates equilibrium restrictions imposed by Le Châtelier′s principle, and the reaction′s negative heat of reaction (ΔH_rxn_) allows ethane ODH to be thermodynamically favorable even at lower reaction temperatures. The process’ oxidative environment also reduces coke formation common in thermal cracking and catalytic dehydrogenation.^13,15^ Co‐feeding C_2_H_6_ and O_2_, however, diminishes the process’ practicality and safety. Downstream product separation results in parasitic losses,^13,15^ and mixing hydrocarbons with oxygen at high temperatures poses an explosion hazard. Chemical looping oxidative dehydrogenation (CL‐ODH) sidesteps these concerns by relying on selective reactive intermediates to facilitate ethane oxidation. In doing so, reducing and oxidizing gases never mix, lessening the demand on downstream cryogenic distillation units and the risk of combustion. Previous experimentation and modeling have highlighted the potential of ethane CL‐ODH to achieve higher ethylene yields than cracking while lowering emissions and energy requirements. Many surface‐promoted mixed metal oxides are active for ethane CL‐ODH under temperatures ranging from 400–850 °C, with the best performing CL‐ODH catalysts to date reaching C_2+_ olefin yields as high as 74 %.^16–25^ Aspen Plus^®^ models of ethane CL‐ODH have also estimated up to an 82 % reduction in energy consumption and CO_2_/NO_x_ emissions relative to steam cracking processes.^26^

Although conventional ODH and CL‐ODH catalysts can significantly enhance olefin yield, they do not inherently offer the capacity to capture and utilize CO_2_. In addition, these catalysts often over‐oxidize olefin products, leading to undesirable CO_x_ selectivities. CO_2_‐mediated ethane ODH (CO_2_‐ODH) has sought to overcome these limitations by enabling CCU in tandem with intensified olefin synthesis.^27–38^ Under this arrangement, carbon dioxide replaces oxygen as the oxidant, directly activating ethane and/or reacting with hydrogen from cracking to improve C_2_H_4_ production. As a weaker oxidant than O_2_, CO_2_ can also reduce instances of C_2_H_6_ overoxidation and enhance ethylene selectivity. Although highly promising, applications of CO_2_‐mediated ethane ODH have faced process inefficiencies related to (i) the need for a pure CO_2_ feed stream, (ii) high reaction endothermicity and equilibrium limitations, and (iii) insufficiently selective catalysts.^39^ Furthermore, while some CO_2_‐ODH catalysts have attained ethylene yields comparable to that of steam cracking, few have simultaneously converted enough CO_2_ to eliminate the need for an additional CO_2_ separation step downstream.^36,40–44^ Therefore, an optimized CO_2_ carrier for CO_2_‐mediated ODH should both improve ethylene production *and* sufficiently convert CO_2_ *in situ*.

**Figure 1:**
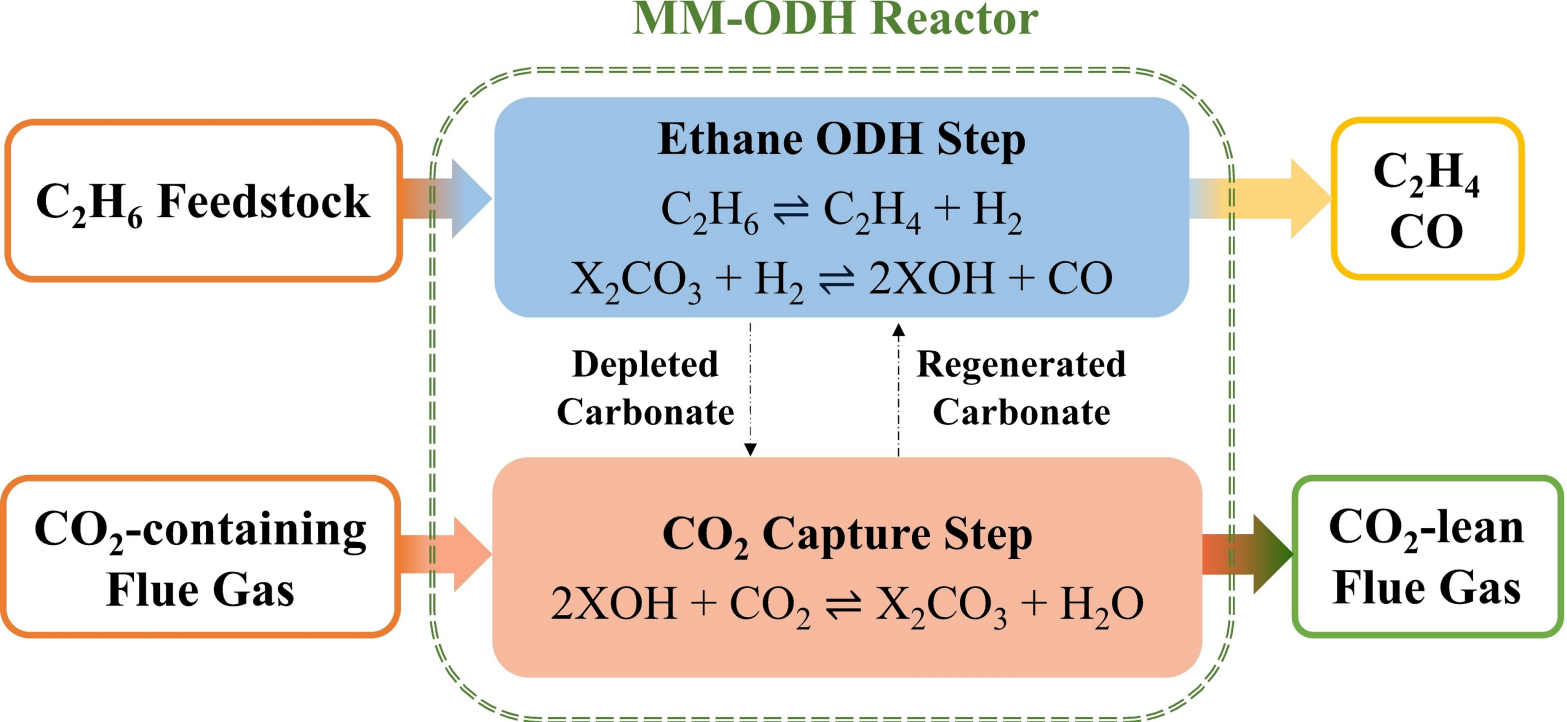
MM‐ODH reaction scheme. X represents Li, Na, and/or K alkali metal cations. Each MM‐ODH cycle consists of an ethane dehydrogenation step and a carbonate regeneration (i. e., CO_2_ capture) step.

In 2021, Liu et al. identified a novel molten‐carbonate‐mediated chemical looping scheme for ethane ODH (MM‐ODH) capable of achieving both high ethylene yields (Y_C2H4_) and CO_2_ conversions (X_CO2_) by segregating ethane and flue gas CO_2_ into gas and melt phases, respectively.^45^ Illustrated in Figure 1, the MM‐ODH configuration acts to boost ethylene yield via a modified reverse water‐gas shift (RWGS) reaction facilitated by the melt, wherein hydrogen produced by ethane pyrolysis interacts with alkali carbonates in the ODH reactor to form alkali hydroxides and CO.^45^ The hydroxides then revert to carbonates by reacting with CO_2_ from flue gas to generate steam during a re‐carbonation step. With carbon monoxide and ethylene co‐produced, many value‐added, low‐carbon‐intensity chemicals can be synthesized with further processing. To our knowledge, this application represented the first instance of molten‐salt‐mediated ethane ODH with *in‐situ* CO_2_ capture *and* utilization.

While the initial work by Liu et al. effectively proved the MM‐ODH concept, limited attention was given to the relationship between carbonate composition and process outcomes, particularly at higher operating temperatures. Therefore, the current work systematically investigated the influence of LNK composition on MM‐ODH performance at 800 °C. Reactive testing of fifteen different LNK mixtures revealed that the MM‐ODH of ethane within Li_2_CO_3_‐rich LNK media can realize ethylene yields greater than those of thermal cracking while simultaneously capturing up to 76.3 % of CO_2_ in a simulated power plant flue gas stream. CO co‐production also exceeded the equilibrium performance of conventional co‐fed CO_2_‐ODH across a wide range of compositions. Li_2_CO_3_‐rich melts produced the most CO on average due partly to contributions from melt‐mediated reforming, whereas near‐eutectic LNK compositions demonstrated the highest modified RWGS activity. When added to Li_2_CO_3_, Na_2_CO_3_ and K_2_CO_3_ attenuated sample decomposition, leading to improved long‐term sample stability and reduced CO_2_ concentrations in the product streams. Finally, an empirical model informed by experimental data and reaction thermodynamics was proposed, which adequately predicted overall MM‐ODH performance from LNK composition alone and provided insights into the system′s primary drivers.

## Results and Discussion

### LNK Composition Study

**Figure 2:**
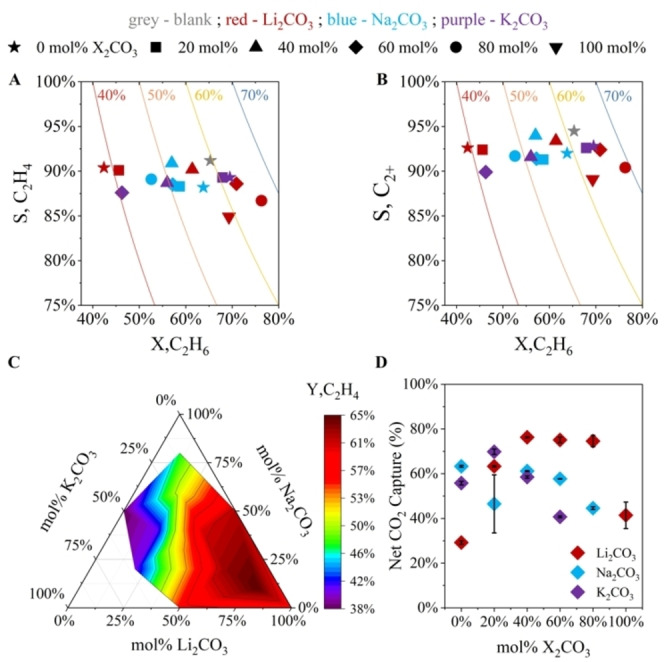
a)–c) MM‐ODH conversion, selectivity, and yield data as functions of alkali carbonate composition in LNK mixtures. d) Net CO_2_ capture from flue gas simulant per MM‐ODH cycle as a function of alkali carbonate composition in LNK. T=800 °C, P=1 bar, gas flow rate during ethane oxidation=45 SCCM (~15 vol% C_2_H_6_, balance Ar), gas flow rate during carbonate regeneration=37.5 SCCM (15 vol% CO_2_, 3 vol% O_2_, balance Ar).

Previous works identified eutectic LNK (43.5 mol % Li_2_CO_3_, 31.5 mol % Na_2_CO_3_, 25 mol % K_2_CO_3_, or 43.5–31.5–25) and a few related binary eutectics as effective MM‐ODH media at ≤770 °C.^45,46^ Because none had related performance to salt composition with enough granularity to formulate a predictive model at higher temperatures, three series of experiments were conducted to systematically determine the respective impacts of Li_2_CO_3_, Na_2_CO_3_, and K_2_CO_3_ content on ethane MM‐ODH at 800 °C. Salt samples were synthesized by maintaining equimolarity between two of the three LNK carbonate constituents while increasing the concentration of the third component, X_2_CO_3_ (X=Li, Na, or K) (Table 1). Starting from 0 mol % X_2_CO_3_, the concentration of X_2_CO_3_ was increased in 20 mol % increments until either a pure carbonate was obtained or the melting point of the resulting salt exceeded 800 °C. Using this approach, fifteen distinct LNK compositions were synthesized and tested. Salt samples in this study were each subjected to 10 MM‐ODH cycles, each of which consisted of a 10‐minute ethane oxidation step and 3‐minute carbonate regeneration step separated by 6‐minute argon (Ar) purges (see Note S1 for details related to the determination of step durations). The reactor effluents from the last three cycles were analyzed via gas chromatography (GC). The average C_2_H_4_ yields, product selectivities, and net CO_2_ capture percentages from these cycles were plotted in Figure 2, with standard error bars indicating their spreads. All compositions showed hydrocarbon mass balances within ±10 % of 100 % after accounting for cracking and reforming products (Table 2), with most samples falling within 5 % (Table S2).

**Table 1:** Compositions of LNK melts subjected to reactive testing.

Sample Number	Li_2_CO_3_ Content (mol %)	Na_2_CO_3_ Content (mol %)	K_2_CO_3_ Content (mol %)
1	**0**	50	50
2	**20**	40	40
3	**40**	30	30
4	**60**	20	20
5	**80**	10	10
6	**100**	0	0
7	50	**0**	50
8	40	**20**	40
9	30	**40**	30
10	20	**60**	20
11	10	**80**	10
12	50	50	**0**
13	40	40	**20**
14	30	30	**40**
15	20	20	**60**

**Table 2:** Ethane MM‐ODH and reaction stoichiometry depicting the primary products and byproducts detected in the reactor effluent via gas chromatography.

MM‐ODH Reactions	
*Reaction*	*Type*
C_2_H_6_ $\vec{\leftarrow }$ C_2_H_4_+H_2_	Ethylene formation via thermal cracking
H_2_+CO_3_^2−^ $\vec{\leftarrow }$ CO+2OH^−^	Modified reverse water‐gas shift

Side Reactions	
*Reaction*	*Type*
C_2_H_6_+2CO_2_ $\vec{\leftarrow }$ 4CO+3H_2_	Melt‐mediated dry reforming
C_2_H_6_+H_2_ $\vec{\leftarrow }$ 2CH_4_	
3 C_2_H_6_ $\vec{\leftarrow }$ 2 C_3_H_6_+3H_2_	Thermal cracking*
2 C_2_H_6_ $\vec{\leftarrow }$ C_4_H_6_+3H_2_	

*Reactions are intended to show stoichiometric relationships only.

The results shown in Figures 2a–c indicated that larger quantities of Li_2_CO_3_ in the melt generally improved ethane conversion (X_C2H6_) and ethylene yield (Y_C2H4_) at the expense of ethylene selectivity (S_C2H4_), attaining their respective maxima of 76.3 % and 64.8 % in the 80–10–10 mixture. Contrarily, increasing the concentrations of Na_2_CO_3_ and K_2_CO_3_ relative to their counterparts adversely impacted X_C2H6_ and Y_C2H4_, though these metrics more notably decreased per 20 mol % increment of K_2_CO_3_ than per increment of Na_2_CO_3_. Despite this trend, S_C2H4_ remained relatively constant across these samples.

Each melt′s capacity for CO_2_ capture largely related to its stability. Because Li_2_CO_3_ decomposed more readily than Na_2_CO_3_ or K_2_CO_3_, Li_2_CO_3_‐rich melts stripped CO_2_ from the flue stimulant most effectively. However, lithium carbonate′s fast rate of decomposition also resulted in these samples rapidly re‐releasing captured CO_2_ during the ethane oxidation and purge steps. Therefore, while 100–0–0 demonstrated consistently high CO_2_ capture capacity throughout its regeneration step, the compositions that demonstrated the highest *net* CO_2_ capture per MM‐ODH cycle contained some stabilizing Na_2_CO_3_ and K_2_CO_3_ (Figures 2d and S2).

**Figure 3:**
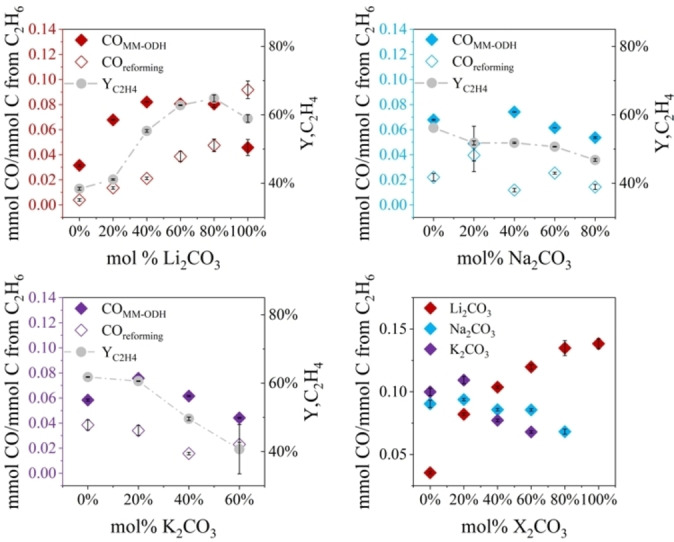
Normalized CO produced via reforming and RWGS activity as functions of a) Li_2_CO_3_, b) Na_2_CO_3_, and c) K_2_CO_3_ content. For most compositions, changes to Y_C2H4_ roughly followed changes to CO_MM‐ODH_. d) Total normalized CO produced via both reforming and RWGS shift activity for all samples. T=800 °C, P=1 bar, gas flow rate during ethane oxidation=45 SCCM (~15 vol% C_2_H_6_, balance Ar), gas flow rate during carbonate regeneration=37.5 SCCM (15 vol% CO_2_, 3 vol% O_2_, balance Ar).

CO co‐production also varied with composition. In most samples, more CO was produced than CO_2_ was captured per steady‐state cycle. This phenomenon highlighted the presence of side reactions resembling dry reforming, which likely occurred either through direct interaction of C_2_H_6_ with LNK or indirectly through melt‐mediated coke gasification.^47–51^ For each sample, the CO contribution from dry reforming was calculated using a normalized CO_2_ mass balance over the complete MM‐ODH cycle (Table S1). All compositions exhibited some extent of dry reforming behavior, although the quantity of CO produced via the modified RWGS reaction (n_CO,MM‐ODH_) was greater than that of reforming (n_CO,reforming_) in every sample except for pure Li_2_CO_3_. n_CO,MM‐ODH_ and n_CO,reforming_ also tended to trend somewhat synchronously in most cases (Figures 3a–c). Beyond 80 mol % Li_2_CO_3_, Y_C2H4_ declined as total CO yield remained monotonic, a phenomenon which further confirmed the presence of multiple CO‐producing reactions.

In summary, while interaction effects unavoidably obscured clear correlations between individual melt constituents and each MM‐ODH performance metric, high Li_2_CO_3_ content generally improved C_2_H_4_ yield, total CO output, and net CO_2_ capture capacity. While Na_2_CO_3_ and K_2_CO_3_ adversely impacted MM‐ODH product yields, their presence served to dampen sample decomposition.

### Extended MM‐ODH Cycling

**Figure 4:**
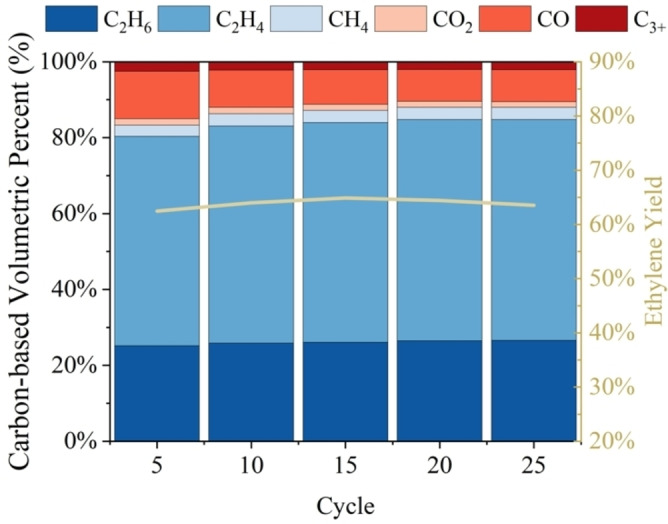
Twenty‐five MM‐ODH redox cycles in 60–20–20 LNK. T=800 °C, P=1 bar, gas flow rate during ethane oxidation=45 SCCM (~15 vol% C_2_H_6_, balance Ar), gas flow rate during carbonate regeneration=37.5 SCCM (15 vol% CO_2_, 3 vol% O_2_, balance Ar).

Having demonstrated favorable stability, Y_C2H4_, net CO_2_ capture, and CO co‐production during the composition study, the 60–20–20 mixture was subjected to extended cycling to assess its long‐term performance. A total of twenty‐five cycles were run on a fresh sample using the same flow rates and conditions described previously. Gas bag samples for GC analysis were collected every five cycles, and mass spectrometry measured the effluent composition of the cycles in between. For the duration of the experiment, the 60–20–20 sample generated a consistent MM‐ODH product distribution and stable Y_C2H4_ (Figure 4). Plumbing inside and downstream of the reactor remained unobstructed by salt deposits for the entirety of the test as well. A separate 60–20–20 sample was also subjected to five hundred cycles of continuous operation, demonstrating similarly stable effluent composition throughout despite a reactor leak precluding accurate calculation of the product yields (Figure S4). Nevertheless, the outcomes from both tests highlighted the durability of the MM‐ODH process under extended operation and its overall promise as a practical CCU application.

### MM‐ODH Mechanistic Investigation

#### Computational Simulation of Direct and Radical‐Based Interactions

**Figure 5:**
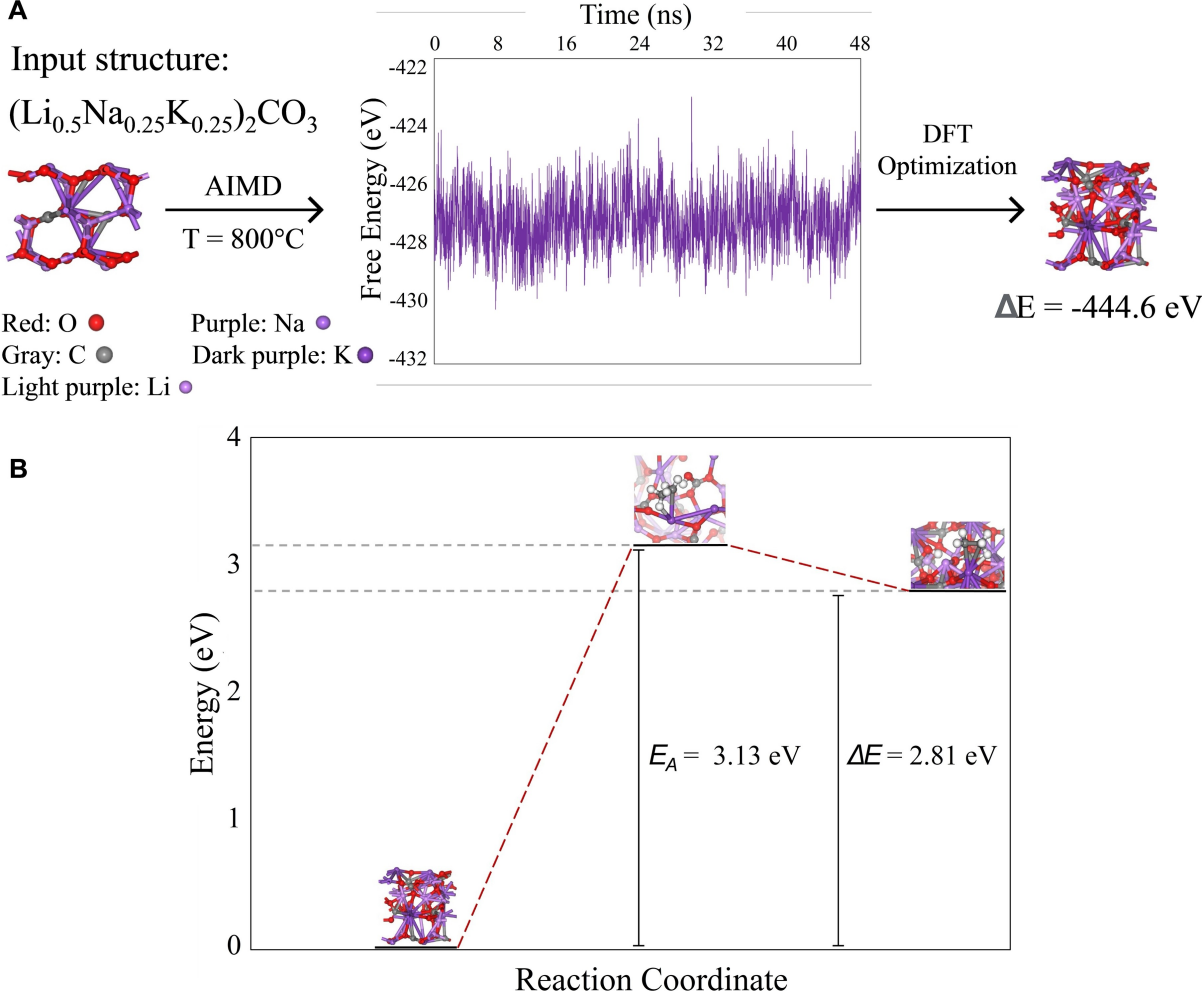
a) AIMD free energy equilibration of the (Li_0.5_Na_0.25_K_0.25_)_2_CO_3_ molten carbonate structure at 800 °C followed by DFT optimization. b) Energy barrier for C_2_H_6_ dehydrogenation.

While LNK composition evidently influenced MM‐ODH performance, the underlying mechanism and role of each melt component in it remained unclear. Therefore, computational and thermodynamic calculations were carried out to gain insight into possible reactive pathways and to formulate a predictive model from the experimental data. To first validate a computational model for exploring gas‐melt chemical interactions, constant temperature AIMD simulations run using the Vienna Ab‐Initio Simulation Package (VASP) investigated the direct activation of ethane by LNK as a test case. A (Li_0.5_Na_0.25_K_0.25_)_2_CO_3_ mixture, which closely resembled the eutectic LNK composition, served as the input structure for AIMD equilibration calculations at 800 °C. After 5 picoseconds (ps), the total energy of the structure stabilized as the melt assumed its lowest energy configuration. From this molecular dynamics trajectory, 10 candidate structures were then selected for further optimization by DFT calculations, with the most stable structure functioning as the input medium for the C_2_H_6_ dehydrogenation reaction. The resulting reaction coordinate for the direct dehydrogenation of C_2_H_6_ by the molten carbonate showed an activation energy (E_a_) of 3.13 eV and a ΔE equal to 2.81 eV, implying that the direct activation of ethane by (Li_0.5_Na_0.25_K_0.25_)_2_CO_3_ was both kinetically and thermodynamically unfavorable (Figure 5). This result was consistent with experimental findings from Liu et al.^45^

**Figure 6:**
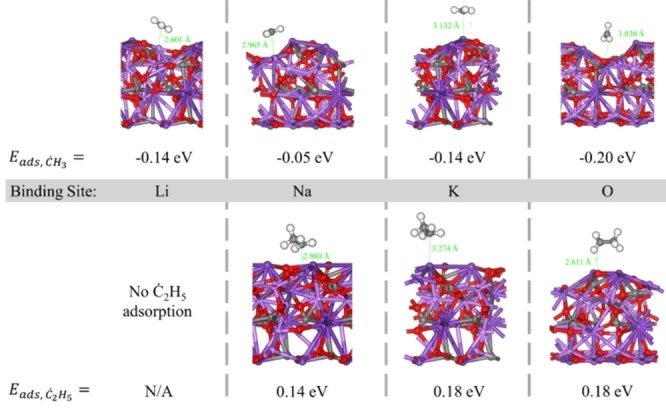
Methyl and ethyl radical interactions with the alkali metal and oxygen surface sites on (Li_0.5_Na_0.25_K_0.25_)_2_CO_3_.

Unfavorable reaction energetics and a low physical solubility of C_2_H_6_ in molten carbonates^46^ have strongly suggested that gas‐phase cracking primarily drives ethane conversion. It is well‐known that this reaction proceeds via a radical pathway whereby the C−H and C−C bonds in C_2_H_6_ break to form ethyl and methyl radicals, respectively. These unstable species then propagate chain reactions before terminating to form hydrocarbon products and hydrogen. Physicochemical interactions at the gas‐melt interface were therefore hypothesized to involve radical hydrocarbon species, with the extent of these interactions relating to LNK composition. Using AIMD and DFT calculations, we simulated the interactions of C_2_H_5_ ⋅  and CH_3_ ⋅  with the Li, Na, K, and O sites of (Li_0.5_Na_0.25_K_0.25_)_2_CO_3_. The energies of adsorption of the two radical species onto each of these sites were then calculated. Small adsorption energies between the radical species and surface sites suggested physisorption, and no notable relationships between adsorption energy and radical distance from the surface were observed (Figure 6). From these calculations, significant hydrocarbon radical adsorption onto any melt constituent was deemed unlikely.

#### Measured MM‐ODH Performance vs. Conventional CO_2_‐ODH Equilibrium

**Figure 7:**
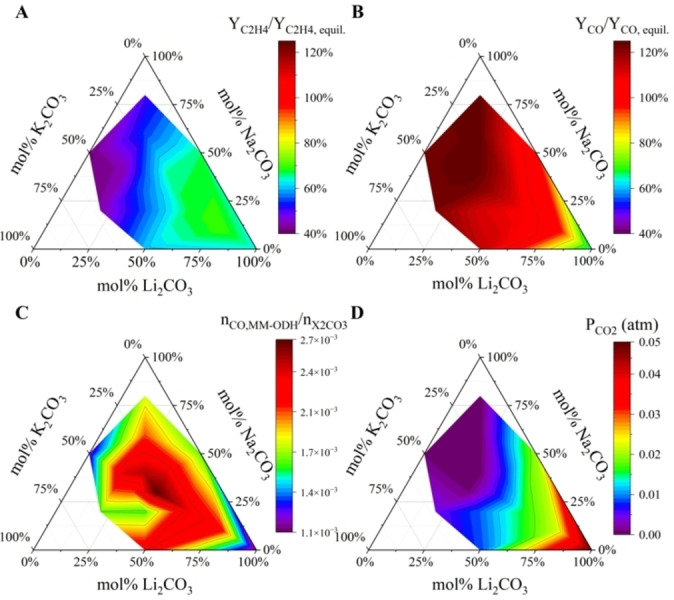
a)–b) Ternary contour plots relating LNK composition to equilibrium‐scaled Y_C2H4_ and Y_CO_. Equilibrium yields were defined as those predicted in an equilibrated, co‐fed CO_2_‐ODH system. c) Carbonate turnover (n_CO,MM‐ODH_/n_X2CO3_) and d) measured partial pressure of CO_2_ (P_CO2_) in the ODH step as functions of LNK composition.

In the absence of direct catalytic ethane activation by LNK, the relationship between MM‐ODH activity, carbonate stability, and ethane cracking was interpreted through a thermodynamic lens. In Liu et al.,^45^ temperature‐programmed reduction (TPR) and H_2_:CO_2_ co‐feed experiments indicated that (1) the MM‐ODH scheme enabled super‐equilibrium CO yields (Y_CO_, defined in Table S1) and (2) thermodynamic, not kinetic, drivers primarily governed ethane cracking reactions. To relate this thermodynamic basis for MM‐ODH to LNK composition, Y_C2H4_ and Y_CO_ for each sample were scaled relative to their expected equilibrium yields under a conventional co‐fed CO_2_‐ODH system and plotted as functions of alkali carbonate content (Figures 7A−B). Equilibrium yields were determined using an extent of reaction (EoR) approach (Note S2). Reforming and C_1_/C_3+_ hydrocarbon reactions, which were predominantly driven by kinetics, were not included in the EoR system of reactions.

As seen in Figures 7A and B, Y_C2H4_ and Y_CO_ reached their respective maxima under different sets of compositions, with their gradients pointing in opposite directions. Ethylene yield most closely approached its equilibrium limit at 80 mol % Li_2_CO_3_, whereas the degree of Y_CO_ super‐equilibrium increased with decreasing Li_2_CO_3_ content. As defined, Y_CO_ directly related to salt stability, so stable LNK mixtures were expected to exhibit larger extents of RWGS super‐equilibrium and thus lower CO_2_ separation demands (Figure 7B and D). The extent of C_2_H_6_ cracking, on the other hand, did not clearly relate to a salt′s tendency to decompose. In fact, Figure 7C indicated that the cracking and modified RWGS reactions operated most synergistically near the eutectic composition, where n_CO,MM‐ODH_ generated per mole of total carbonate was highest. This efficient carbonate conversion suggested that near‐eutectic LNK melts converted H_2_ most effectively. Because this region did not coincide with that of peak cracking activity, the modified RWGS reaction did not appear to be the primary driver of ethylene production.

To test the predictive capability of the four ternary plots shown in Figure 7, two previously untested sample compositions, 15–34–51 and 70–22–8, were randomly generated and subjected to MM‐ODH cycling. Equilibrium‐scaled product yields were calculated from GC data for both samples and compared to their predicted values derived from the shown contour plots. Considering the small sample size relative to the design space, the predicted equilibrium ratios for both 15–34–51 and 70–22–8 adequately approximated the equilibrium ratios and modified RWGS activity obtained from experimentation (Table 3). Although the predicted P_CO2_ for the 70–22–8 sample closely matched its experimental value, the two values largely differed for the stable 15–34–51 sample in terms of the P_CO2_ value. This large discrepancy likely arose from errors in integrating very small, partially obscured CO_2_ GC peaks (Figure S5) and does not impact the overall qualitative trend in this region.

**Table 3:** Predicted vs. actual values for C_2_H_4_ and CO equilibrium ratios, RWGS activity, and P_CO2_. The notation x mol % Li_2_CO_3_ – y mol % Na_2_CO_3_ – z mol % K_2_CO_3_ indicates each sample′s composition.

	15–34–51		70–22–8	
Metric	Predicted	Actual	Predicted	Actual
$\frac{Y_{C_{2}H_{4}}}{Y_{C_{2}H_{4},equil.}}$	42 %	35.4 %±0.0	68 %	68.5 %±0.0
$\frac{Y_{CO}}{Y_{CO,equil.}}$	121 %	124 %±0.1	98 %	99.7 %±3.3
$\frac{n_{CO,MM-ODH}}{n_{X_{2}CO_{3}}}$	1.77x10^−3^	1.96x10^−3^	2.01x10^−3^	2.24x10^−3^
$P_{CO_{2}}$ (atm)	.002	2.8x10^−4^	.020	2.2x10^−2^

#### Role of LNK Oxobasicity in the MM‐ODH Mechanism

Solvated oxyanions (e. g., O^2−^, O_2_^2−^, O_2_^−^ , $CO_{4}^{2-}$ ), which have been extensively investigated in molten LNK electrochemistry, represented another set of transient chemical species which may have participated in aspects of the MM‐ODH mechanism. In Lux‐Flood acid‐base chemistry,^52–54^ the relative concentrations of oxide (O^2−^) donors (i. e., oxobases) and acceptors (i. e., oxoacids) determine a medium′s overall acid‐base reactivity, with molten carbonates representing highly oxobasic media due to carbonate decomposition.

$$CO_{3}^{2-}\vec{\leftarrow }CO_{2}+O^{2-}$$

While appreciable concentrations of O^2−^ exist in LNK melts, the anion′s full valence shell makes it unlikely to oxidize ethane. For an oxide‐assisted cracking mechanism to exist, electrophilic oxygen species would have to be sufficiently present under MM‐ODH conditions. Voltammetry, electron spin resonance (ESR), and *in situ* Raman studies have previously identified $CO_{4}^{2-}$ , $O_{2}^{2-}$ , and $O_{2}^{-}$ in high‐temperature alkali and alkaline earth carbonate melts, but such detection usually occurred under electrochemical conditions and/or atmospheres with high P_O2_.^48,55–74^ Consequently, oxyanions are unlikely to participate in C_2_H_6_ conversion in MM‐ODH, which is carried out under an O_2_‐free environment. Melt oxobasicity did likely influence CO_2_ uptake, however. The magnitude of this impact will be explored as part of a future mechanistic study.

## Conclusions

This work evaluated the performance of fifteen molten LNK media in the MM‐ODH of ethane, supporting the feasibility of this new process scheme as a sustainable alternative to steam cracking for ethylene production. Nine of the fifteen LNK mixtures generated ethylene yields comparable to that of thermal cracking while capturing up to 76.3 % of CO_2_ from a simulated power plant flue gas to co‐produce CO. Both ethylene yield and net CO_2_ capture attained their maxima (64.8 % and 76.3 %, respectively) in melts consisting primarily of Li_2_CO_3_. Relatively smaller concentrations of Na_2_CO_3_ and K_2_CO_3_ in these samples enhanced their overall stability. The inherent separation of C_2_H_6_ and CO_2_ facilitated by LNK also resulted in super‐equilibrium CO production relative to conventional CO_2_‐ODH across nearly all compositions, with samples rich in Na_2_CO_3_ and K_2_CO_3_ realizing the highest degrees of super‐equilibrium. This large CO/CO_2_ ratio in the MM‐ODH product stream significantly reduces the need for downstream CO_2_ separation and is a defining feature of the scheme.

Computational and thermodynamic approaches provided insight into the mechanism(s) and overall drivers of MM‐ODH performance. Mixtures near the eutectic composition were found to most efficiently convert carbonates into CO via the modified RWGS reaction. Because near‐eutectic compositions did not coincide with those which realized the highest Y_C2H4_, the modified RWGS reaction was determined to not be the primary driver behind melt‐mediated improvements to ethane conversion. AIMD and DFT calculations suggested that direct C_2_H_6_ activation or hydrocarbon radical interactions at the gas‐melt interface were unlikely. Ethane oxidation by peroxide or superoxide species was also deemed improbable, although melt oxobasicity likely played an important role in CO_2_ capture activity. Measured MM‐ODH C_2_H_4_ and CO yields were scaled relative to conventional CO_2_‐ODH equilibrium yields to develop an empirical correlation between LNK compositions and MM‐ODH performance. Ternary contour plots generated from these scaled values accurately predicted the MM‐ODH performance of two randomly generated LNK compositions, validating the usefulness of the proposed correlation to guide the design and optimization of the molten salt medium for MM‐ODH applications.

## Experimental Section

### Redox Catalyst Syntheses and MM‐ODH Reactive Testing

**Figure 8:**
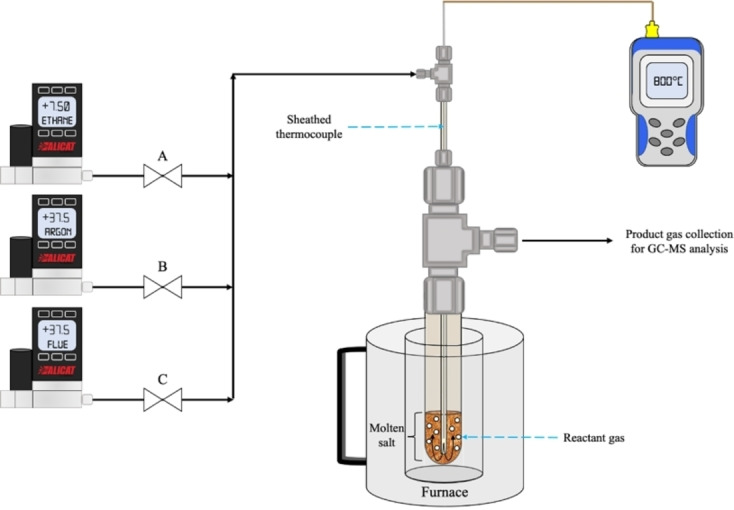
Schematic of MM‐ODH lab‐scale experimental system.

The ternary Li_2_CO_3_‐Na_2_CO_3_‐K_2_CO_3_ salt mixtures were synthesized according to the phase diagram presented in Janz et al.^75^ Prior to each experiment, anhydrous Li_2_CO_3_ (Sigma Aldrich, ≥99.0 % purity), Na_2_CO_3_ (Sigma Aldrich, ≥99.5 % purity), and K_2_CO_3_ (Sigma Aldrich, ≥99 % purity) precursors were physically mixed in their desired molar ratios to a total mixture mass of 20 g. These mixtures were then transferred to closed‐end alumina reactor tubes (McDanel Ceramics, OD: 1.000+/−0.050”, ID: 0.750+/−0.038”, LG: 7.000+/−0.063”). After fitting a loaded ceramic tube to a Swagelok tee with PTFE ferrules (Figure 8), the tube was then suspended in a high‐temperature vertical furnace and heated at a rate of 20 °C/min until the salt reached the MM‐ODH reaction temperature of 800 °C.

Alicat mass flow controllers (MFCs) and electrically actuated Emerson ASCO solenoid valves modulated gas flow into the ionic melt through a 1/4” O.D. ceramic injection tube. The injection tube housed a 1/16” sheathed thermocouple and bubbled the gas phase through the molten mixture (Figure 8). Prior to MM‐ODH cycling, a flue gas simulant supplied by Airgas (15 % CO_2_, 3 % O_2_, balance Ar) was continuously injected into each melt until full carbonate saturation was achieved. Each MM‐ODH cycle consisted of a 10‐minute ethane oxidation step and a 3‐minute carbonate regeneration step separated by 6‐minute purges with Ar. During ethane oxidation, the MFCs charged 7.5 SCCM of C_2_H_6_ and 37.5 SCCM of Ar to the reactor. During carbonate regeneration, 37.5 SCCM of flue simulant re‐carbonated the salt prior to starting the next cycle. 37.5 SCCM of Ar purged the system between each step. Each MM‐ODH cycle operated isothermally and isobarically at 800 °C and ~1 bar, respectively. An in‐line MKS Cirrus III quadrupole mass spectrometer carried out residual gas analysis (RGA) of the reactor effluent to determine real‐time gas compositions. Gas samples for GC analysis were collected in Tedlar gas bags after the system attained steady cycle‐over‐cycle performance, with each collection spanning the entirety of the oxidation or regeneration step and 5 minutes of the following purge. These samples were analyzed using an Agilent 7890B gas chromatograph equipped with a flame ionization detector (FID) and two thermal conductivity detectors (TCD). OpenLab EZChrom software executed residence‐time‐based peak identification, with further analysis done using Microsoft Excel and OriginLab.

To obtain a thermal cracking blank, the column heights of two fully dense salts in their respective ceramic tubes were measured. A clean ceramic tube was then filled with white α‐Al_2_O_3_ powder from Kramer Industries, Inc., (16 mesh, packing density≈60 %) to a height 1.67 times that of the salts’ average (~28 g). This scaling ensured equivalent void volumes between the experimental and blank tests. Minimal variation in column height among all fused LNK samples also allowed for sufficiently accurate approximation of their average using only two samples. The Al_2_O_3_ blank was finally heated to 800 °C and subjected to the cycling conditions described previously to obtain thermal cracking data as an MM‐ODH performance reference.

### Computational Methods

DFT calculations within periodic slabs were carried out using VASP.^76–79^ The generalized gradient approximation (GGA) method calculated electronic structures using a PBE functional,^80^ and the projector‐augmented wave (PAW) method described electron‐core interactions.^81,82^ The energy cutoff and force threshold for all calculations were set to 450 eV and 0.05 eV/Å, respectively, and a 3‐layer, 1×2 Li_2_CO_3_(001) supercell with a vacuum layer greater than 12 Å was constructed. To build a structure comparable to Li_0.6_Na_0.2_K_0.2_CO_3_ from this supercell, Li atoms were randomly substituted with Na and K atoms to create a Li_0.5_Na_0.25_K_0.25_CO_3_ ternary salt. A 2×2×1 k‐point grid described the surface Brillouin zone.

AIMD calculations in the NVT ensemble on the (Li_0.5_Na_0.25_K_0.25_)_2_CO_3_ surface were modeled by conducting structural relaxations at 800 °C for at least 50 ps, a setting used in previous works.^83,84^ Each system consisted of 80 atoms in 2 nm^2^ of space, and the structures’ total energies equilibrated after 5 ps. Following the 50 ps relaxation period, 10 random structures from the final 10 ps underwent full DFT‐driven structural optimization (Figure S3). The arrangements with the most stable energies among the 10 optimized candidates were selected to serve as input structures for the modeling of dehydrogenation reactions between the salt structures and gaseous reactants. The adsorption energies were calculated as *E*_ads_=*E*_adsorbate+slab_−(*E*_adsorbate_+*E*_slab_), where *E*_adsorbate+slab_ comprised the energies of the slab and one adsorbed species, *E*_adsorbate_ represented the energy of the species in the gas phase, and *E*_slab_ was the energy of the slab structure. To evaluate the adsorption energy trends with increasing surface coverage of a species, the cumulative adsorption energy was used and calculated as *E*_cum._(n)=*E*_*n*,ads.+slab_−*E*_*n−1*,ads.+slab_−*E*_adsorbate+slab+_ *E*_slab_. Here, *E*_*n*,ads.+slab_ and *E*_*n−1*,ads.+slab_ denote the energies of the surface structure with n and n−1 adsorbed species (n≥2), respectively. For all the structures, the bottom half of the atoms were relaxed during calculation. Transition state structures were obtained by the CI‐NEB method and characterized via vibrational frequency calculations.

## Supporting Information Summary

The authors have cited additional references within the Supporting Information (Ref. [86–89]).

## Conflict of Interests

The authors declare no conflict of interest.

1

## Supporting information

As a service to our authors and readers, this journal provides supporting information supplied by the authors. Such materials are peer reviewed and may be re‐organized for online delivery, but are not copy‐edited or typeset. Technical support issues arising from supporting information (other than missing files) should be addressed to the authors.

Supporting Information

## Data Availability

The data that support the findings of this study are available from the corresponding author upon reasonable request.
