# Supplementary material for: Molten‐Salt‐Mediated Chemical Looping Oxidative Dehydrogenation of Ethane with In‐Situ Carbon Capture and Utilization
Source: ChemSusChem. 2024 Nov 15;18(6):e202401473. doi: 10.1002/cssc.202401473 (PMC11912109; doi:10.1002/cssc.202401473)
Supplement: Supplementary file 1 — Supporting Information [file CSSC-18-e202401473-s001.pdf]

ChemSusChem

Supporting Information

Molten-Salt-Mediated Chemical Looping Oxidative Dehydrogenation of Ethane with *In-Situ* Carbon Capture and Utilization

Kyle Vogt-Lowell, Dennis Chacko, Kunran Yang, Jace Carsten, Junchen Liu, Matthew Housley, and Fanxing Li*

ChemSusChem

Supporting Information

Molten-Salt-Mediated Chemical Looping Oxidative Dehydrogenation of Ethane with *In-Situ* Carbon Capture and Utilization

Kyle Vogt-Lowell, Dennis Chacko, Kunran Yang, Jace Carsten, Junchen Liu, Matthew Housley, and Fanxing Li*

Supporting Information for

Molten-salt-mediated Chemical Looping Oxidative Dehydrogenation of Ethane with In-situ Carbon Capture and Utilization

Kyle Vogt-Lowell, Dennis Chacko, Kunran Yang, Jace Carsten, Junchen Liu, Matthew Housley†, Fanxing Li*

Department of Chemical and Biomolecular Engineering, North Carolina State University, 911 Partners Way, Raleigh, North Carolina, 27695-7905

†*School of Engineering, Newcastle University, Merz Court, Newcastle upon Tyne NE1 7RU, United Kingdom*

Number of Pages: 16

Number of Figures: 6

Number of Tables: 6

Notation

$n_{C_2H_6,in}$	–	moles of C_2H_6 injected during the ethane oxidation step
$n_{C_2H_6,out}$	–	moles of C_2H_6 in the effluent product stream
$n_{C_xH_y}$	–	moles of C_xH_y in the effluent product stream
$n_{total\ C,out}$	–	total moles of C in the effluent product stream
$n_{CO_2,in}$	–	moles of CO_2 injected during the carbonate regeneration step
$n_{CO_2,out\ (red.)}$	–	moles of CO_2 in the effluent product stream during the ethane oxidation step (carbonate reduction)
$n_{CO_2,out\ (purge)}$	–	moles of CO_2 in the effluent product stream during both purge steps
$n_{CO_2,out\ (regen)}$	–	moles of CO_2 in the effluent product stream during the regeneration step
$n_{CO,total}$	–	total moles of CO in the effluent product stream during the ethane oxidation step
s	–	sample standard deviation

Table S1. Equations for MM-ODH reaction parameters.

Parameters	Equations
Ethane Conversion (X_{C2H6})	$\frac{n_{C_2H_6,in} - n_{C_2H_6,out}}{n_{C_2H_6,in}}$
C_xH_y Selectivity (S_{CxHy})	$\frac{(x)(n_{C_xH_y})}{n_{total\ C,out} - (2)(n_{C_2H_6,out}) - (x)(n_{C_xH_y}) - n_{CO_2,out\ (red.)} - n_{CO,MM-ODH}}$
C_2H_4 Yield (Y_{C2H4})	$X_{C_2H_6} \cdot S_{C_2H_4}$
C_{2+} Selectivity (S_{C2+})	$\frac{n_{total\ C,out} - n_{CH_4} - (2)(n_{C_2H_6,out}) - n_{CO_2,out\ (red.)} - n_{CO,total}}{n_{total\ C,out} - (2)(n_{C_2H_6,out}) - n_{CO_2,out\ (red.)} - n_{CO,MM-ODH}}$
CO from MM-ODH ($n_{CO,MM\text{-}ODH}$)	$n_{CO_2,in} - n_{CO_2,out\ (regen)} - n_{CO_2,out\ (purge)} - n_{CO_2,out\ (red.)}$
CO from reforming ($n_{CO,reforming}$)	$n_{CO,total} - n_{CO,RWGS}$

Net CO_2 capture	$\frac{n_{CO,MM-ODH}}{n_{CO_2,in}} \cdot 100\%$
CO Yield (Y_{CO})	$\frac{n_{CO,MM-ODH}}{n_{CO,MM-ODH} + n_{CO_2,out\ (red.)}} \cdot 100\%$
Standard Error	$\frac{s}{\sqrt{n}}$, where n represents the number of MM-ODH cycles

Table S2. Average MM-ODH performances for tested ternary salt compositions.

Composition x – y – z (x mol% Li_2CO_3 – y mol% Na_2CO_3 – z mol% K_2CO_3)	X_{C2H6}	Y_{C2H4}	S_{C2H4}	S_{C2+}	S_{CH4}	Net CO_2 Capture	Reduction Carbon Balance (%)
Blank	65.3%	59.5%	91.2%	94.5%	4.8%	--	100 ± 1.5
0 – 50 – 50	42.4%	38.4%	90.4%	92.6%	6.9%	29.2%	99 ± 0.5
20 – 40 – 40	45.6%	41.1%	90.1%	92.4%	6.3%	63.3%	102 ± 0.3
40 – 30 – 30	61.4%	55.3%	90.2%	93.4%	4.9%	76.3%	104 ± 1.0
60 – 20 – 20	70.8%	62.8%	88.6%	92.4%	4.8%	75.1%	98 ± 1.7
80 – 10 – 10	76.3%	64.8%	86.7%	90.4%	6.3%	74.6%	104 ± 1.9
100 – 0 – 0	69.3%	58.9%	84.9%	89.1%	4.8%	41.4%	104 ± 1.1
50 – 0 – 50	63.8%	56.2%	88.2%	92.0%	6.1%	63.3%	94 ± 0.4
40 – 20 – 40	58.7%	51.8%	88.3%	91.3%	5.7%	46.5%	110 ± 0.9
30 – 40 – 30	57.0%	51.8%	90.9%	94.0%	4.9%	61.2%	101 ± 0.6
20 – 60 – 20	57.2%	50.7%	88.5%	91.4%	6.5%	57.7%	100 ± 0.1
10 – 80 – 10	52.6%	46.8%	89.1%	91.7%	6.9%	44.6%	100 ± 0.6
50 – 50 – 0	69.5%	61.8%	89.3%	92.8%	4.4%	55.8%	103 ± 0.5
40 – 40 – 20	67.9%	60.6%	89.3%	92.6%	4.7%	69.8%	94 ± 0.6
30 – 30 – 40	56.0%	49.6%	88.7%	91.6%	7.1%	58.5%	102 ± 1.2
20 – 20 – 60	46.3%	40.7%	87.6%	89.9%	7.6%	40.7%	101 ± 1.4

Prior to each experiment, a handheld leak detector was used to check for leaks on the MM-ODH reactor plumbing. A DryCal Defender flow meter was then used to verify gas flows at room temperature prior to temperature ramping.

The calculations shown below were used to determine the hydrocarbon mass balance during the ethane oxidation step of each MM-ODH cycle. Here, only half of the CO produced from dry reforming can be attributed to hydrocarbons.

$$C\ balance = \frac{mmol\ C\ out}{mmol\ C\ in}$$

$$mmol\ C\ in = (n_{C_2H_6,in})(2\ mol\ C/1\ mol\ C_2H_6)$$

$$mmol\ C\ out = \left(\sum_{i=1}^{5}(n_{C_iH_{2i+2}})(i) + \sum_{j=2}^{5}(n_{C_jH_{2j}})(j) + 2n_{C_2H_2} + 3n_{C_3H_4} + 4n_{C_4H_6} + 6n_{C_6H_6}\right) + \frac{n_{CO,reforming}}{2}$$

Table S3. Reactions considered in carbon balancing.

**Reaction**	**Reaction Type**	**Hydrocarbon Product**
$C_2H_6 + H_2 \rightleftharpoons 2CH_4$	Thermal cracking	methane
$C_2H_6 \rightleftharpoons C_2H_4 + H_2$		ethylene
$C_2H_6 \rightleftharpoons C_2H_2 + 2H_2$		acetylene

$3C_2H_6 \rightleftharpoons 2C_3H_8 + H_2$	(Note: Thermal cracking reactions are intended to show the stochiometric relationships only.)	propane
$3C_2H_6 \rightleftharpoons 2C_3H_6 + 3H_2$		propylene
$3C_2H_6 \rightleftharpoons 2C_3H_4 + 5H_2$		propadiene<br>methyl acetylene
$2C_2H_6 \rightleftharpoons C_4H_{10} + H_2$		butane
$2C_2H_6 \rightleftharpoons C_4H_8 + 2H_2$		butene
$2C_2H_6 \rightleftharpoons C_4H_6 + 3H_2$		1,3-butadiene
$5C_2H_6 \rightleftharpoons 2C_5H_{12} + 3H_2$		pentane
$5C_2H_6 \rightleftharpoons 2C_5H_{10} + 5H_2$		pentene
$3C_2H_6 \rightleftharpoons C_6H_6 + 6H_2$		benzene
$C_2H_6 + 2CO_2 \rightleftharpoons 4CO + 3H_2$	Melt-mediated dry reforming	-
$H_2 + CO_2 \rightleftharpoons CO + H_2O$	Reverse water-gas shift	-

Note S1. Selection of MM-ODH Cycle Step Durations

Ethane Oxidation

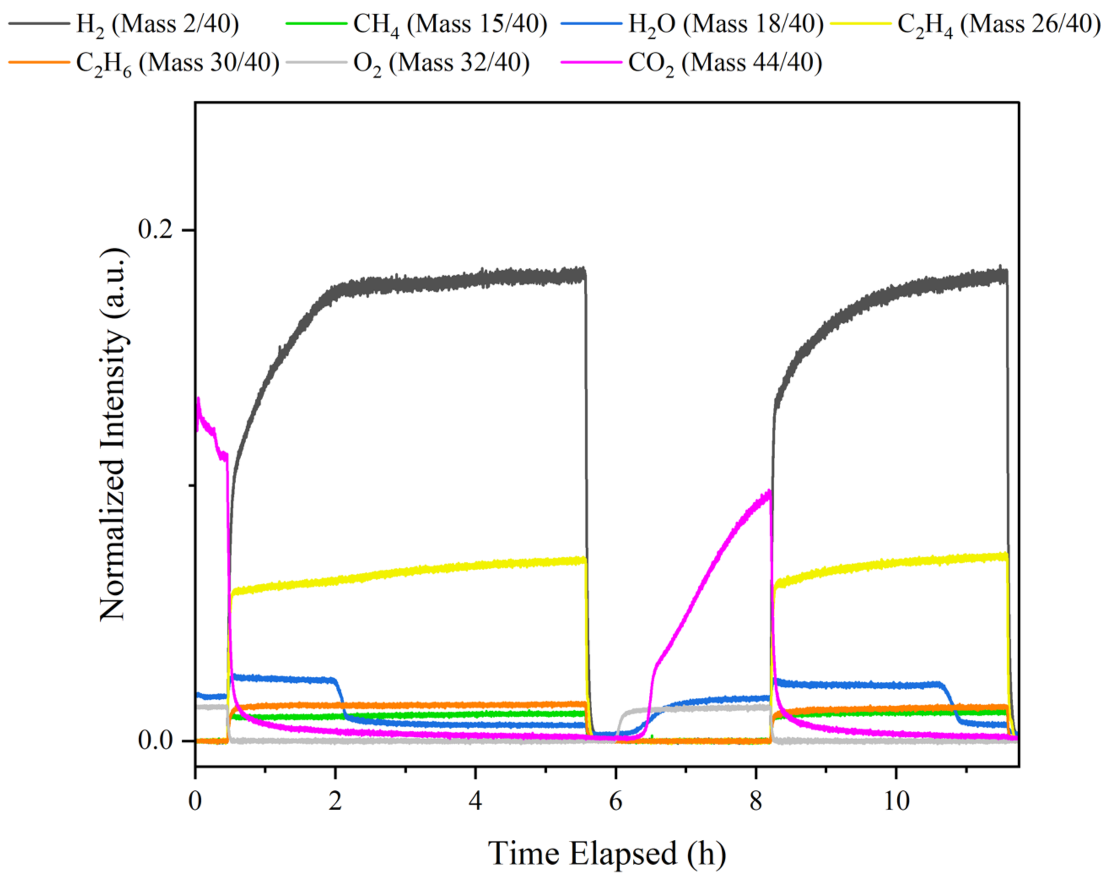

Figure S1. Two extended injections of C_2H_6 (15 vol%, balance Ar) into 80 – 10 – 10 LNK at T = 800 C, P = 1 atm. Between the extended ODH steps, the carbonate was regenerated to ensure equivalent levels of salt saturation prior to each C_2H_6 injection.

The selection of the ODH step duration was informed by previous experimental data. Additionally, such cycle durations are consistent with the commercially proven, cyclic CATOFIN process for propylene production.

The first set of MM-ODH experiments were those performed by Liu et al. (2020)[46] during the initial proof-of-concept. There, C_2H_6 injections lasted for 4 minutes. To assess the extent to which this duration could be extended, 15 vol% ethane (balance Ar) was injected into the MM-ODH reactor (T = 800°C, P = 1 atm, 20 g 80-10-10 LNK) continuously for several hours without regenerating the molten carbonate. The resulting mass spectroscopy (MS) data (**Figure S1**) showed ethylene yield to increase only marginally after hours of injection. In practical applications, however, a prolonged ODH step without melt regeneration can result in excessive carbonate decomposition, leading to more volatile alkali metal oxide/hydroxide species. To mitigate this concern and align the MM-ODH operating conditions with those of existing commercial processes, the final ODH step duration was set to 10 minutes, a length which approximately matched the ODH step duration of the CATOFIN process[86].

Inert Gas Purge

To reduce the overall MM-ODH cycle time and mitigate LNK decomposition, the 6-minute duration of each purge step was chosen to closely represent the time required to sufficiently remove reactive gases from the reactor between the ODH and regeneration steps for the current reactor setup.

Carbonate Regeneration

The length of the regeneration was set to 3 minutes to ensure both sufficient carbonate regeneration and moderate-to-high CO_2 capture during the regeneration step across several LNK compositions. **Figure S2** illustrates the carbon capture activity of four LNK mixtures during their fifth regenerations as functions of time. The 100 – 0 – 0, 10 – 80 – 10, and 0 – 50 – 50 compositions represent the extreme cases of Li_2CO_3, Na_2CO_3, and K_2CO_3 content, respectively, whereas 60 – 20 – 20 represents an intermediate case.

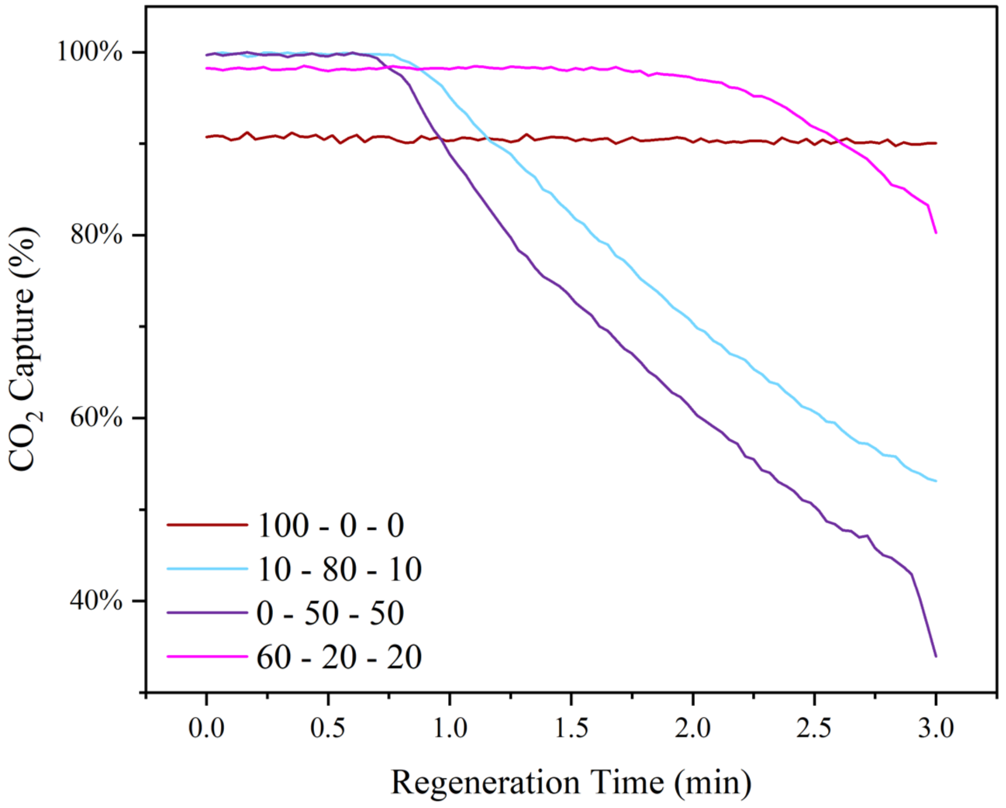

Figure S2. CO_2 capture activities of 100 – 0 – 0, 10 – 80 – 10, 0 – 50 – 50, and 60 – 20 – 20 LNK as functions of regeneration time. Each sample was subjected to five MM-ODH cycles, with the above data representing CO_2 uptake behavior during their final regeneration.

Note S2. Extent of Reaction and Equilibrium Calculations

Table S4. Variables used in extent of reaction and equilibrium calculations.

Variable	Definition
$K_{1,\ 800°C}$	C_2H_6 thermal cracking equilibrium constant at 800°C
$K_{2,\ 800°C}$	Reverse water-gas shift equilibrium constant at 800°C
ξ_1	C_2H_6 thermal cracking extent of reaction
ξ_2	Reverse water-gas shift extent of reaction
y_A	Mole fraction of species A
n_{A_0}	Initial moles of species A
n_0	Total initial moles of all chemical species
$Y_{C_2H_4,\text{equil.}}$	Equilibrium yield of C_2H_4 at 800°C
$Y_{CO,\text{equil.}}$	Equilibrium yield of CO at 800°C

System of Reactions

(I) $C_2H_6 \rightleftharpoons C_2H_4 + H_2$ $K_{1,\ 800°C} = 1.170$

(II) $H_2 + CO_2 \rightleftharpoons H_2O + CO$ $K_{2,\ 800°C} = 0.9326$

Extent of Reaction Relationships

$$y_{C_2H_6} = \frac{n_{C_2H_{6_0}} - \xi_1}{n_0 + \xi_1}$$

$$y_{CO_2} = \frac{n_{CO_{2_0}} - \xi_2}{n_0 + \xi_1}$$

$$y_{C_2H_4} = \frac{\xi_1}{n_0 + \xi_1}$$

$$y_{CO} = y_{H_2O} = \frac{\xi_2}{n_0 + \xi_1}$$

$$y_{H_2} = \frac{\xi_1 - \xi_2}{n_0 + \xi_1}$$

$$K_{1,800°C} = \frac{(y_{C_2H_4})(y_{H_2})}{y_{C_2H_6}} = \frac{\xi_1(\xi_1 - \xi_2)}{(n_0 + \xi_1)(n_{C_2H_{6_0}} - \xi_1)} \qquad \boldsymbol{\xi_1 = 2.856}$$

$$K_{2,800°C} = \frac{(y_{CO})(y_{H_2O})}{(y_{CO_2})(y_{H_2})} = \frac{(\xi_2)^2}{(n_{CO_{2_0}} - \xi_2)(\xi_1 - \xi_2)} \qquad \boldsymbol{\xi_2 = 0.551}$$

The above two equations were subjected to the following constraints and solved using Excel Solver and MATLAB:

- $\xi_1 - \xi_2 \neq 0$
- $n_0 + \xi_1 > 0$
- $\mathrm{n}_{\mathrm{C_2H_6}_0} - \xi_1 > 0$
- $\mathrm{n}_{\mathrm{CO_2}_0} - \xi_2 > 0$
- $0 \leq y \leq 1$

Because $\xi_1 = \mathrm{n}_{\mathrm{C_2H_4}}$and $\xi_2 = \mathrm{n}_{\mathrm{CO,MM-ODH}}$ according to the extent of reaction relationships, $Y_{C2H4,equil.}$ and $Y_{CO,equil.}$ were calculated as follows:

$$\mathrm{Y}_{\mathrm{C_2H_4,equil.}} = \frac{\xi_1}{\mathrm{n}_{\mathrm{C_2H_6}_0}} \cdot 100\% = 92.9\% \qquad \mathrm{Y}_{\mathrm{CO,equil.}} = \frac{\xi_2}{\mathrm{n}_{\mathrm{CO_2}_0}} \cdot 100\% = 80.4\%$$

Note S3. Bubble Size Approximation

Bubble size represents an important MM-ODH process parameter for two primary reasons. First, the extent of C_2H_6 conversion via thermal cracking is directly related to gas holdup in the reactor. While the volume of gas in the reactor headspace was approximately constant, the volume of each injected bubble could change with salt composition and thereby impact performance. Second, the interfacial surface area of each injected gas bubble directly relates to the rate of mass transfer across the gas-melt interface. This relationship is analytically expressed as:

$$\dot{n}_m = kA\Delta c_m$$

$\dot{n}_m$ – mass transfer rate of species m
k – mass transfer coefficient
A – effective mass transfer area
Δc_m – concentration difference of species m

In a bubble column, the interfacial surface area is a function of a bubble's ascending velocity and diameter[87]. The impact of LNK composition on ascending velocity in this system was indirectly deemed negligible via the described MRT experiments. Further investigation was needed, however, to correlate LNK composition to bubble size.

van Krevelen and Hoftijzer[87] proposed a theoretical hydrostatic force balance to relate bubble volume to surface tension, melt density, and gas density. For systems using low flow rates and small injection orifices, the hydrostatic equation could predict bubble volume (V) within a factor of two[87,88]. While quantitatively useful in some cases, the other assumptions built into this equation precluded its use for accurate quantification in our system.

$$\frac{g(\rho - \rho_g)\delta^3}{\gamma d} = \text{constant} \approx 6$$

$$V = \frac{\pi d\gamma}{(\rho - \rho_g)g}$$

g – acceleration due to gravity
ρ – melt density
ρ_g – gas density
δ – bubble diameter
γ – surface tension
d – orifice diameter of gas injector

Qualitatively, however, the relationship did highlight melt surface tension as the predominant factor in determining MM-ODH bubble size, given the high surface tension of molten LNK. Therefore, the surface tensions of six LNK compositions previously characterized by Janz et al.[89] were compared to gauge the value's range across compositions similar to those tested experimentally. For each calculated surface tension value, a weighted average gas density of ρ_g = 0.0004 g/mL was used for 16% C_2H_6 in Ar at approximately 800°C.

Table S5. Melt surface tensions and densities derived from Janz et al. for six LNK compositions.[89]

Composition (mol% Li_2CO_3 – mol% Na_2CO_3 – mol% K_2CO_3)	Surface Tension, γ (dyne/cm)	γ Uncertainty	Melt Density, ρ (g/mL)	ρ Uncertainty
0 – 50 – 50	186.6	0.5%	1.964	0.5%
43.5 – 31.5 – 25	212.5	0.5%	1.920	1%
100 – 0 – 0	242.2	1.5%	1.803	0.5%

50 – 0 – 50	187.2	3%	1.872	0.5%
50 – 50 – 0	227.9	1.5%	1.897	0.5%
20 – 80 – 0	235.3	1.5%	1.970	0.5%

Table S5 showed the largest difference in surface tension among these compositions to be $\Delta\gamma$ = 55.60 dyne/cm. With the MM-ODH reactor design preventing the direct observation of bubbles in molten 0 – 50 – 50 and 100 – 0 – 0, a simple room temperature experiment was carried out instead to assess bubble size in two liquids with a similar $\Delta\gamma$. In this experiment, Ar was bubbled through two glass beakers, one containing water (γ = 73.6 dyne/cm) and one ethanol (γ = 22 dyne/cm), at a flow rate of 45 SCCM through the same injection tube used during reactive testing. Slow motion videography recorded the bubble frequencies over three 10-second intervals in each beaker, and the average bubble frequencies in both water and ethanol were calculated. The total injected volume of gas over the 10-second injection interval was then divided by these average frequencies to approximate a gas volume per bubble. As shown in **Table S6**, no notable difference in bubble volume between the two liquids was observed, so the tested salt compositions were assumed to have roughly uniform bubble sizes.

Table S6. Bubble volume data for room-temperature water and ethanol experiments.

**Liquid** (T ≈ 25 °C)	Ar Flow Rate (SCCM)	Surface Tension (γ) (dyne/cm)	Average Bubble Frequency (s^{-1})	Bubble Volume (mL)
Water	45	73.6	3.1 ± 0.1	0.24 ± 0.01
Ethanol	45	22	3.2 ± 0.1	0.23 ± 0.01

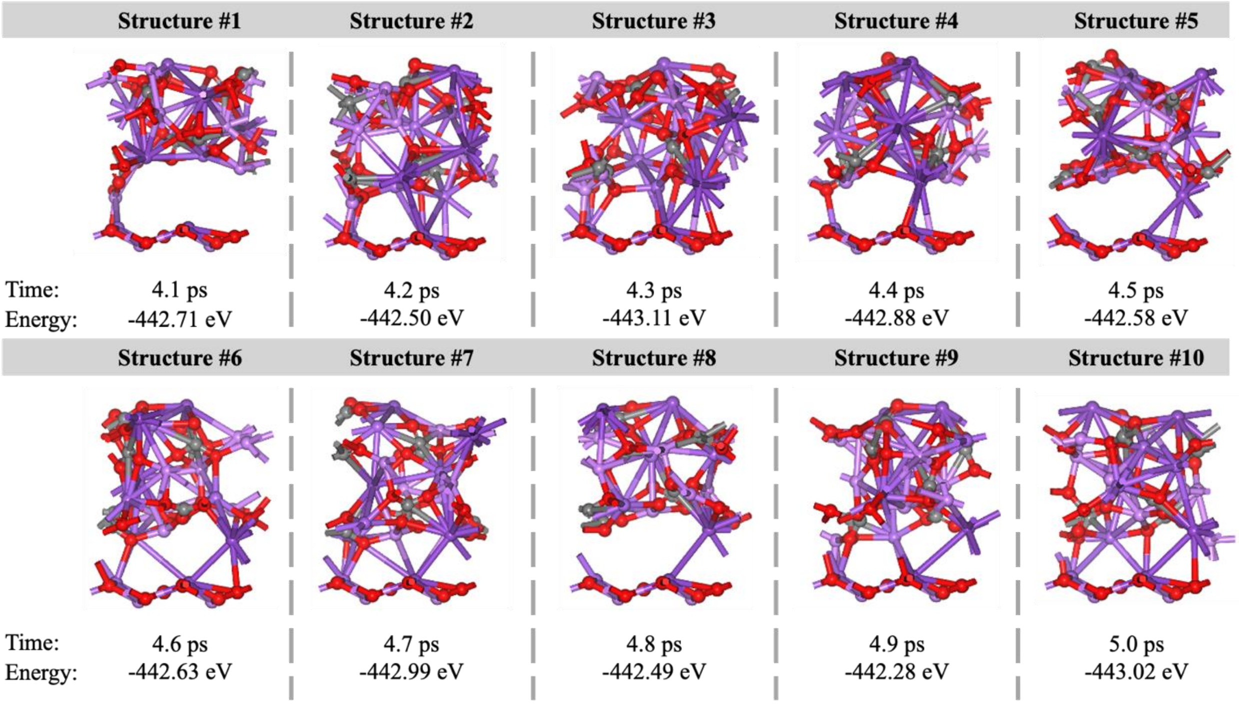

Figure S3. Structure snapshots of $Li_{0.5}Na_{0.25}K_{0.25}CO_3$ from AIMD simulation trajectory. These structures were selected for further optimization via DFT. Time values correspond to the time elapsed since the start of the 5-picosecond equilibration window, and energy values indicate the DFT energy of each structure following structural optimization.

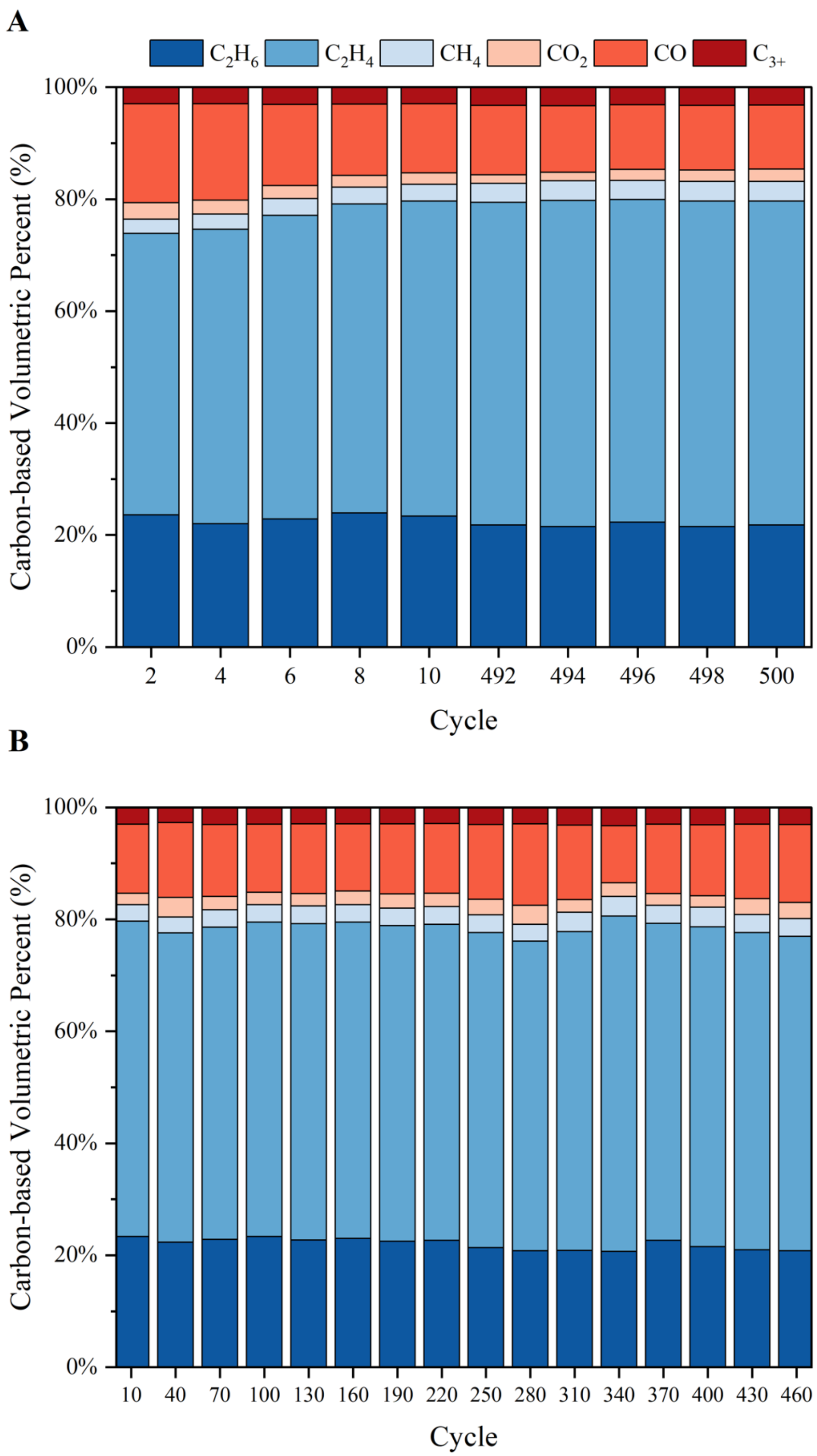

Figure S4. MM-ODH reactor effluent composition over 500 continuous cycles of 60 – 20 – 20 LNK. The durations of ethane oxidation and carbonate regeneration in the first and last 10 cycles were 10 minutes and 3 minutes, respectively. For cycles 10 – 460, these durations were reduced to 3 minutes and 1 minute, respectively. Although a

leak in the reactor during this experiment resulted in carbon balances below 50% for each cycle, the average composition of the effluent stream remained consistent with those of the 60 – 20 – 20 samples that showed satisfactory mass balances.

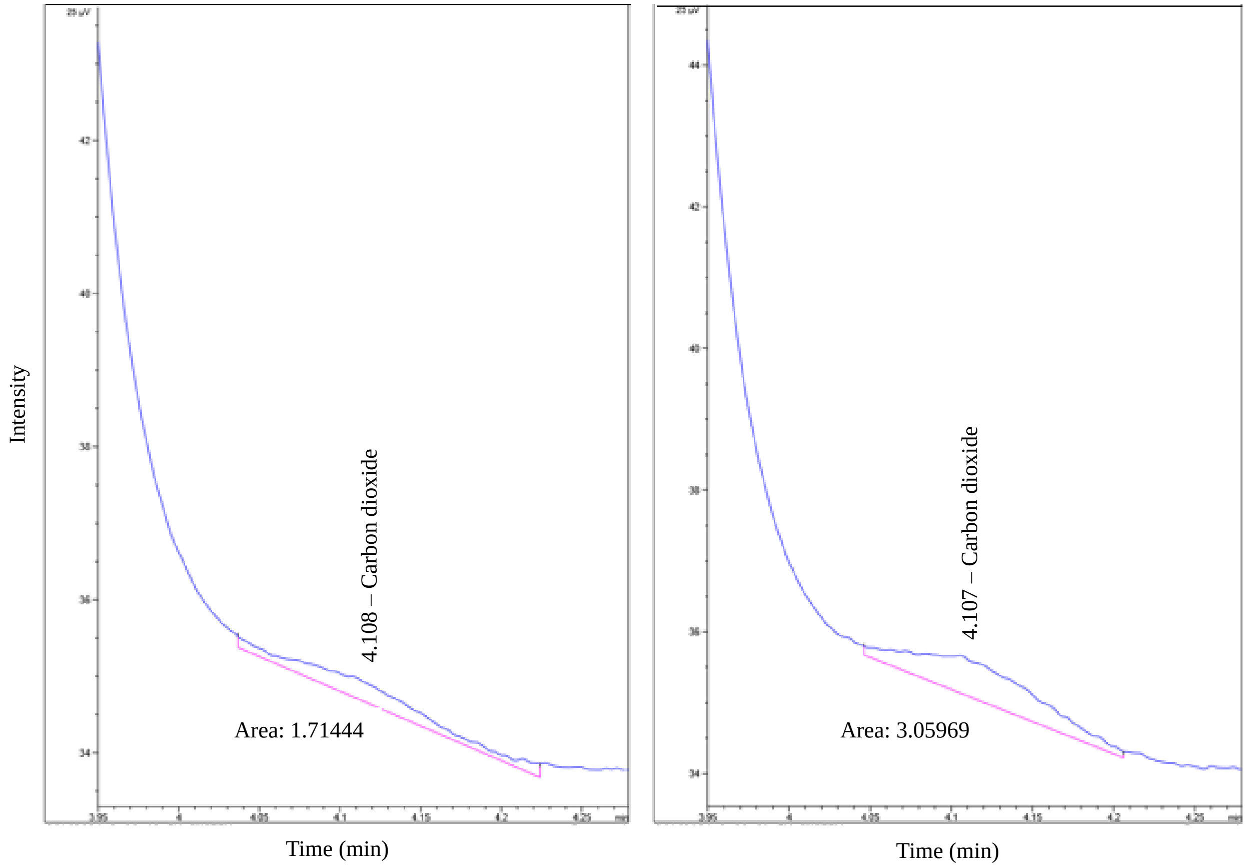

Figure S5. Snapshots of GC CO_2 signals measured during ethane oxidation in 15 – 34 – 51 LNK. Convolution of the small CO_2 peak by a large neighboring signal may have resulted in underestimation of the true peak area. This underestimation was likely the source of the large observed discrepancy between the predicted and measured P_{CO2} in the 15 – 34 – 51 sample.

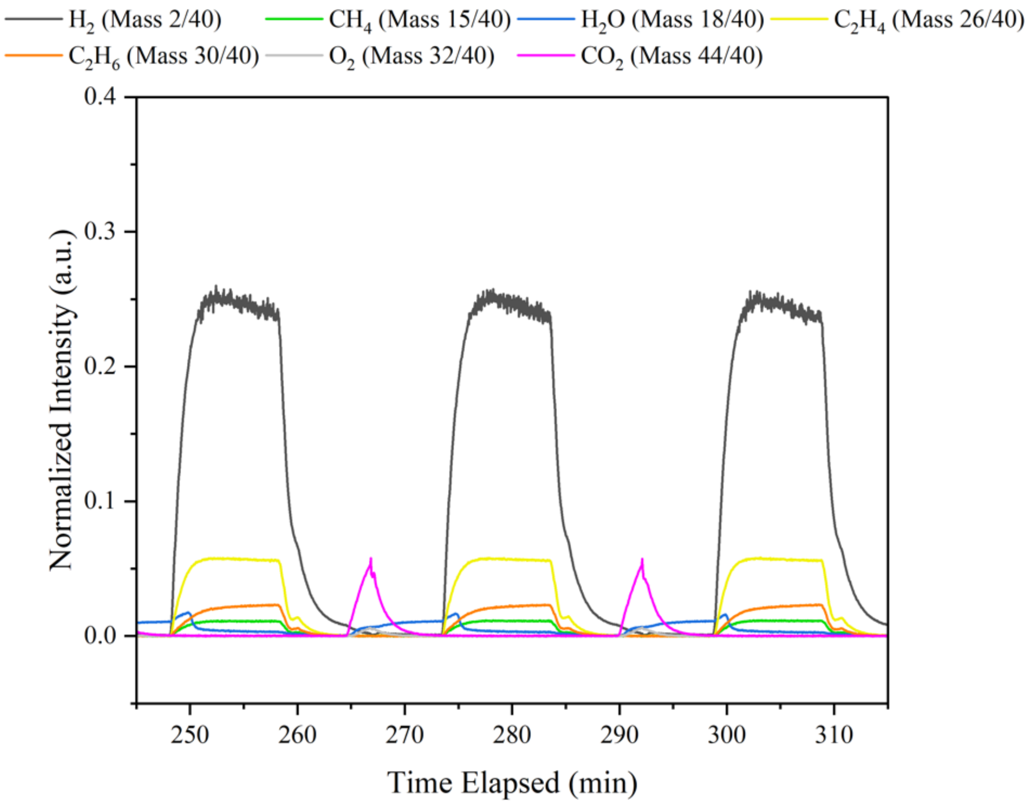

Figure S6. A representative mass spectrum displaying the transient effluent gas composition during MM-ODH cycling of 60 – 20 – 20 LNK. All signal intensities are normalized to the Ar signal (intensity = 1.0; not visible at shown scale). Two and a half MM-ODH cycles are shown, with each full cycle consisting of one ethane oxidation step, one melt regeneration step, and purging intervals in between. During the regeneration, notable interaction between gaseous oxygen from the flue simulant and the molten carbonate was not observed, so all injected O_2 appeared in the effluent. CH_4 appeared as an expected by-product of thermal cracking under every tested LNK composition. Selectivity towards CH_4 varied somewhat with salt composition (4.4 – 7.6% per Table S2, with an overall average of 5.9%). The average methane selectivity over six blank cycles was 4.8%.
